# Morpho-Functional Consequences of *Swiss Cheese* Knockdown in Glia of *Drosophila melanogaster*

**DOI:** 10.3390/cells10030529

**Published:** 2021-03-02

**Authors:** Elena V. Ryabova, Pavel A. Melentev, Artem E. Komissarov, Nina V. Surina, Ekaterina A. Ivanova, Natalia Matiytsiv, Halyna R. Shcherbata, Svetlana V. Sarantseva

**Affiliations:** 1 Molecular and Radiation Biophysics Division, Petersburg Nuclear Physics Institute Named by B.P.Konstantinov of NRC «Kurchatov Institute», 188300 Gatchina, Russia; ryabova_ev@pnpi.nrcki.ru (E.V.R.); melentev_pa@pnpi.nrcki.ru (P.A.M.); komissarov_ae@pnpi.nrcki.ru (A.E.K.); surina_nv@pnpi.nrcki.ru (N.V.S.); katya-i.ivanova@yandex.ru (E.A.I.); 2 Department of Genetics and Biotechnology, Faculty of Biology, Ivan Franko Lviv National University, 79005 Lviv, Ukraine; nataliya.matiytsiv@lnu.edu.ua; 3 Institute of Cell Biochemistry, Hannover Medical School, Carl-Neuberg-Strasse 1, 30625 Hannover, Germany; Shcherbata.Halyna@mh-hannover.de

**Keywords:** *swiss cheese*, *NTE*, *PNPLA6*, *Drosophila melanogaster*, glia, blood–brain barrier, nervous system, ROS, oxidative stress

## Abstract

Glia are crucial for the normal development and functioning of the nervous system in many animals. Insects are widely used for studies of glia genetics and physiology. *Drosophila melanogaster* surface glia (perineurial and subperineurial) form a blood–brain barrier in the central nervous system and blood–nerve barrier in the peripheral nervous system. Under the subperineurial glia layer, in the cortical region of the central nervous system, cortex glia encapsulate neuronal cell bodies, whilst in the peripheral nervous system, wrapping glia ensheath axons of peripheral nerves. Here, we show that the expression of the evolutionarily conserved *swiss cheese* gene is important in several types of glia. *swiss cheese* knockdown in subperineurial glia leads to morphological abnormalities of these cells. We found that the number of subperineurial glia nuclei is reduced under *swiss cheese* knockdown, possibly due to apoptosis. In addition, the downregulation of *swiss cheese* in wrapping glia causes a loss of its integrity. We reveal transcriptome changes under *swiss cheese* knockdown in subperineurial glia and in cortex + wrapping glia and show that the downregulation of *swiss cheese* in these types of glia provokes reactive oxygen species acceleration. These results are accompanied by a decline in animal mobility measured by the negative geotaxis performance assay.

## 1. Introduction

The *swiss cheese* (*sws*) gene of *Drosophila melanogaster* is evolutionarily conserved and its orthologues are found in a wide range of organisms, from bacteria to mammals [1]. Mutations in the *swiss cheese* orthologue gene in humans—*NTE*—lead to different diseases, including organophosphate-induced delayed neuropathy (OPIDN); hereditary spastic paraplegia type 39; pure cerebellar ataxia; ataxia with spasticity; and rare syndromes, such as Gordon Holmes, Boucher–Neuhäuser and Oliver McFarlane syndromes [2,3,4,5]. The *sws* gene encodes a protein with phospholipase B activity that is involved in phosphatidylcholine metabolism [6]. The SWS protein can also function as a regulator of the PKA-C3 catalytic subunit of protein kinase A [7,8]. Mutations in *sws* lead to age-dependent neurodegeneration, the structural alteration of glia cells, and a reduced insect life span [9,10]. SWS is widely expressed in nerve and glial cells [9,10]. The specific subtypes of glial cells that require SWS were formerly identified [9,11]. However, the role of SWS in glial cells that form barriers in the *Drosophila* nervous system is not fully clear.

Unlike the vascularized brain of vertebrates, the nervous system of invertebrates is surrounded by hemolymph, which transfers nutrients and factors through an organism. The selective transfer of various molecules from extracellular fluid to the nervous system is performed by blood barriers: The blood–brain barrier (BBB) in the central nervous system (CNS) and blood–nerve barrier (BNB) in the peripheral nervous system (PNS). Both barriers of *Drosophila melanogaster* cover the whole volume of the nervous system, protecting it from high ion concentrations, especially potassium, an elevated level of which can disrupt regulated electrical conductance [12,13]. The barrier of insects, both functionally and morphologically, resembles the cellular barrier of simple vertebrates [12,14,15]. 

Both barriers of *Drosophila* consist of two sheet-like types of surface glia: perineurial (PG) and subperineurial (SPG) [16,17,18]. The first one is characterized by small sized cells and prolonged nuclei [8]. The specific function of PG has not yet been identified, but PG seems to maintain the integrity of BBB [18]. Additionally, during the larval stage, PG cells continuously divide and form intercellular junctions among themselves, thereby forming a layer covering the SPG [19]. Therefore, it is assumed that PG somehow influences the development and/or tightness of SPG [15]. PG cells resemble mammalian plasmatic astrocytes that are known to form a specific structure filling a big area of capillaries with its processes [14]. The PG layer also performs a barrier function, as large molecules, such as 500 kDa dextran can be stopped by the outer layer of the surface glia [15]. 

The functional SPG barrier forms very early, during embryogenesis [15]. The SPG population is small and represents only 2% of all glia in the organism [18]. It is formed of big square-shaped polyploid cells, and their growth occurs during insect development accompanied by both endomitosis and endoreplication. Importantly, both of these processes ensure a lifelong BBB function and integrity [20]. For instance, the increased ploidy observed under *Ntan1* dysfunction leads to nerve bulge formation accompanied by defasciculated axons in peripheral nerves in the 3rd instar larvae. A high Ntan1 level is necessary for the restriction of the cell cycle and replication rates during larval brain development [21]. This indicates that there is a correlation between the polyploidization level, cell size, and number of nuclei of SPG during insect development. SPG cells form septate junctions that are crucial for the SPG barrier function [22,23,24,25]. It was previously shown that there are some proteins that influence septate junctions of the *Drosophila* cellular barrier, and these factors are vital for BBB development at the embryonic stage. For instance, the dysfunction of claudin-like proteins, such as sinuous and pickle, alters the BBB permeability [15]. 

**Figure 1:**
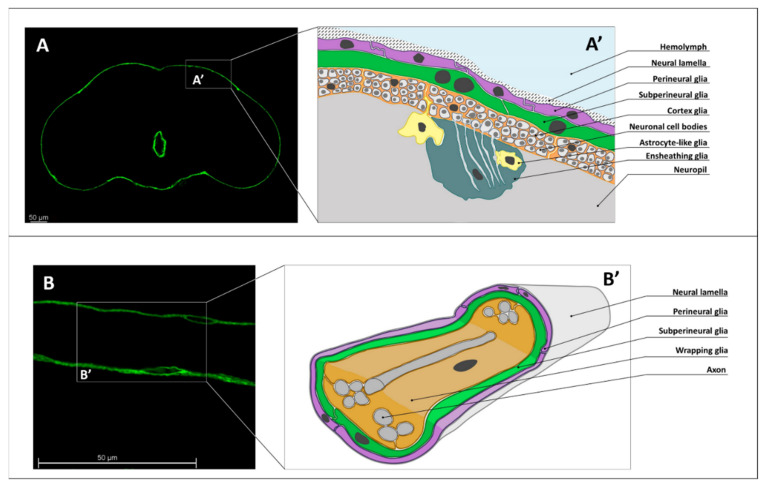
Types of glia in the *Drosophila melanogaster* nervous system. (**A**) Visualization of the *Drosophila* subperineurial glia (SPG) in the central nervous system (CNS) (confocal microscopy image of a brain, where SPG is marked with green fluorescent protein (GFP) in the *NPS-GAL4;UAS-CD8-GFP* genotype). (**A’**) Scheme of the principal *Drosophila* brain cellular structure though a cross-section view, where SPG is shown in green, PG is shown in purple, WG is shown in orange. (**B**) Visualization of the *Drosophila* subperineurial glia in the peripheral nervous system (PNS) (confocal microscopy image of an abdominal nerve, where SPG is marked with GFP in the *NPS-GAL4;UAS-CD8-GFP* genotype). (**B’**) Scheme of the principal *Drosophila* peripheral nerve cellular structure through a three-dimensional cross-section view, where SPG is shown in green, PG is shown in purple, WG is shown in orange. Scale bar: 50 µm.

In the central nervous system, cortex glia (CG) form the cell layer that lies under SPG (Figure 1A). CG function similarly to astrocytes in mammals [19]. In insects, CG cells encapsulate neurons, thereby insulating them from each other and forming a honeycomb-like cortex structure. Furthermore, CG ensheath neuronal processes before intersection of the neuropil [18]. In addition, CG provide neurons with nutrients and gaseous substances. A single CG cell can serve up to 100 neuron bodies [15]. SPG and CG interact tightly with each other through adherens junctions. It has been shown that it is the CG layer that interacts with SPG and accumulates a great amount of lipid droplets [26]. 

In peripheral nerves, wrapping glia (WG) are located under the SPG layer (Figure 1B). Their function in *Drosophila* is similar to that of mammalian non-myelinating Schwann cells. WG maintain neuronal nutrition and help to reduce electric noise between individual axons and prevent ephaptic coupling. Several WG cells can already be detected during embryonic development. After embryogenesis, WG cells begin to differentiate [27]. Disturbances in the development of WG affect the normal functioning of the nervous system [28].

Various genes have been found to regulate the survival and functioning of different glia types [21,29,30,31]. Among them, *swiss cheese* (*sws*) was shown to be important for SPG and ensheathing glia in the CNS neuropile. The knockdown (KD) of *sws* in SPG resulted in CNS vacuolization and locomotor activity disturbance [11]. Additionally, it was initially shown that in newly enclosed *sws* mutant flies, glia undergo hyperwrapping [9,32]. 

In this study, we analyzed the role of *sws* in CNS and PNS glia subtypes at several stages of *Drosophila melanogaster* development.

## 2. Materials and Methods

### 2.1. Drosophila Stocks 

To downregulate *sws*, an RNA-interference-induced approach was used to knock-down (KD) *sws* gene expression in different types of glial cells (*UAS-sws-RNAi*, BDSC 61338): panglially, *repo-GAL4* (BDSC 7415); in perineurial glia, *NP6293-GAL4* (hereinafter *NPP-GAL4*, KYOTO DGGR 105188); in subperineurial glia, *NP2276-GAL4* (hereinafter *NPS-GAL4*, KYOTO DGGR 112853); and in both cortex and wrapping glia, *NP2222-GAL4* (hereinafter *NPC-GAL4*, KYOTO DGGR 112830). Auxiliary fly stocks were used to visualize glia using membrane GFP (*UAS-CD8-GFP*, BDSC 8746) and DsRed protein with nuclear localization (*UAS-RedStinger*, BDSC 8547). Additionally, we used own promotor of *sws* to activate GAL4 expression in cells where *sws* is expressed (*sws-GAL4*, KYOTO DGGR 104592). *UAS-NTE* stock (kindly donated by Robert Wessells) was used to induce human *NTE* gene expression in the rescue experiment. All flies were kept at +25 ˚C in a standard semolina-yeast-sugar medium.

This study was approved by the Ethical Committee of the Petersburg Nuclear Physics Institute named by B.P. Konstantinov of NRC «Kurchatov Institute» (protocol # 01/KПБ of 13 January 2020).

### 2.2. Nervous System Preparation for Confocal Microscopy 

Dissection was performed in phosphate-buffered saline (PBS), and larvae were fixed in 4% paraformaldehyde (PanReac AppliChem, Spain) for 20 min. Then, samples were washed three times in PBS for 5 min and stored in an antifade mounting medium for fluorescent samples (Vectashield, Vector Laboratories, USA). 

Brain and ventral nerve cords of adult flies were dissected and fixed in freshly prepared 4% paraformaldehyde for 10 min. Next, they were washed in PBS 3 × 5 min and placed in a medium for fluorescent microscopy (Vectashield, Vector Laboratories, USA).

### 2.3. Imaging and Analysis

Series of images (2-μm-thick) were obtained with a Leica LX laser confocal microscope (Leica microsystems, Germany) using 20× (air) and 63× (oil) objectives. A quantitative analysis was performed using the ImageJ software (NIH, USA). For a quantitative nuclei calculation and morphological analysis of glial cells, 25 flies were analyzed per genotype.

Confocal images were processed with ImageJ. The standard method for calculating the particle size was used. Three-dimensional (3D) reconstructions of pictures were created in Leica LasX software. The minimal size of 10 μm^2^ was selected for identifying normal SPG nuclei. For PG and CG, a minimal size of 3 μm^2^ was selected. Figures and illustrations were prepared in Leica LasX and Adobe Photoshop CC 2019 (Adobe, USA) (Supplementary Figure S1). For an analysis of small SPG nuclei, which we propose are fragmented, a minimal size of 5 μm^2^ was chosen for particle quantification (Supplementary Figure S2). 

### 2.4. Immunohistochemistry

Dissected brains were fixed for 40 min in 4% paraformaldehyde at room temperature (RT), washed 3 × 5 min in PBS, and stained overnight at +4 °C with primary anti-repo (8D12, Developmental Studies Hybridoma Bank (DSHB), USA) antibodies diluted in blocking buffer (1:50). After washing them in PBS three times, samples were stained for 2 h at RT with a secondary Cy3 antibody (Sigma-Aldrich, USA, 1:200 dilution in blocking buffer).

### 2.5. Survival Analysis 

One to two-day-old imagoes were placed in test tubes with agar and yeast suspension and were kept at +25 °C (30–40 males per vial, with a total of at least 300 males per genotype). Live flies were flipped to a new vial with a fresh medium every 2–3 days and the number of dead individuals was counted. The experiment was conducted until the death of the last fly. 

### 2.6. Preparation and Analysis of Paraffin Sections 

The total neurodegeneration level was assessed in paraffin brain sections. Flies were fixed for 24 h in freshly prepared 4% paraformaldehyde. Then, they were transferred through solutions of 70%, 95%, and 100% ethanol and methyl ester of benzoic acid (Vecton, Russia) and were finally embedded in molten paraffin (Merk, Germany). The obtained fly brain paraffin sections (6-μm-thick) were stained with hematoxylin (BioVitrum, Russia) and eosin (BioVitrum, Russia) after deparaffinization. The degree of neurodegeneration was evaluated as the ratio of the total area of vacuoles to an entire area of a brain section (nine flies per genotype and age, with three good-quality sections per fly, resulting in 27 sample values) using a Leica DM 2500 (Leica microsystems, Germany) light microscope and the ImageJ software. 

### 2.7. Negative Geotaxis Assay 

Locomotor activity and gravity taxis were tested using the RING assay, as described in [33]. Briefly, six groups of 20–40 flies of each genotype and age were transferred into empty vials without anesthesia, and the vials were loaded into the RING apparatus. The apparatus was rapped three times in rapid succession to initiate a negative geotaxis response. The flies’ movements in tubes were videotaped and digital images captured 3 s after initiating the behavior. The distance between a fly and a vial bottom was calculated for each fly. The performance of flies was analyzed in six consecutive trials (interspersed with a 60 s rest). For each genotype and age, more than 200 flies participated in the experiment, and for each fly, six replicate values were obtained, so the total sample size was more than 1200 values.

### 2.8. Learning Indices Assessment 

About 20–30 males per vial were kept until the standard protocol for the olfactory learning test was applied [34,35]. Briefly, all experiments were performed in a temperature-controlled dark room using T-maze, with one odorant (3-octanol or 4-methylcyclohexanol (Fluka, Sigma-Aldrich, Germany)) having been compiled with 12 electric stimuli (60 V) for 1 min during training, while the other odorant had not. After a pause of 2 min, flies were allowed to go into two tubes of T-maze, each containing one of the odorants, for 2 min. Finally, the number of flies that stayed in each tube was counted and the half-index was calculated for each vial, as described in [34]. For both tests with octanol and 4-methylcyclohexanol, nine biological replicates were analyzed (i.e., nine vials with 20–30 flies for each of the odorants). Then, we generated 81 independent pairs of learning half-indices, resulting in a sample size of 81 means (learning indices *per se*). For each age and genotype, more than 600 flies participated in the experiment.

### 2.9. Oxidative Particle Measurement 

The reactive oxygen species (ROS) level was measured using 2’,7’-dichlorodihydrofluorescein diacetate (H2DCFDA, Invitrogen, USA), as described in [36]. Twenty heads for each biological replicate among nine total replicates were homogenized in a buffer containing 100 μL of 10 mM Tris and 3 μL protease inhibitor (Roche, Germany) (pH 7.4). The homogenate was centrifuged (for 10 min at 10,000 rpm, +4 °C). Then, 5 μL of clear supernatant was mixed with 60 μL of 5 μM H2DCF-DA and incubated for 60 min at +37 °C, and the procedure was repeated three times to obtain three technical replicates for each biological replicate. The fluorescence emission of DCF resulting from DCF-DA oxidation was scanned at 485 nm excitation and 530 nm emission with a plate reader (EnSpire2300, PerkinElmer, USA). The values obtained for the ROS levels were normalized to the protein concentration measured by the standard protocol of the Bradford assay and were then averaged for each of the seven biological replicates.

### 2.10. Oxidized Glutathione Relative Level Assessment 

To assess the relative level of oxidized glutathione in the brain glia cytoplasm, special sensor stock was used (*UAS-cyto-Grx1-roGFP2*, kindly donated by Jörg Großhans), as described here [37,38]. Briefly, 12 male brains were incubated in 20 μM N-ethyl maleimide (NEM) (Sigma-Aldrich, USA) for 10 min. Then, they were washed in PBS (BioVitrum, Russia), fixed in 4% formaldehyde solution for 10 min, washed in PBS, and finally placed in Antifade Mounting Medium (Vectashield, Vector Laboratories, USA). The same numbers of brains were preliminarily incubated for 10 min with 100 μM diamide (DA) (Sigma-Aldrich, USA) to oxidize the sensor or 10 μM dithiothreitol (DTT) (Sigma-Aldrich, USA) to reduce the sensor and brains were then washed in PBS. All brain samples were analyzed with a Leica LX laser confocal microscope using a 20× (oil) objective at 405 or 488 nm excitation and 500–530 nm emission, resulting in a series of images (total thickness of 100 μm, and distance between images of 2 μm). A quantitative analysis of the total fluorescence in 3D reconstructed images was performed with ImageJ software (function: total gray area). The criterion for analysis was calculated as the normalized 405/488 ratio of the average fluorescence.

### 2.11. Transcriptomic Assay

Total RNA was extracted from twenty 30-day-old males with the RNeasy Mini Kit (QIAGEN, Germany) with two replicates per genotype, and equal amounts of purified good-quality RNA were then prepared for hybridization using the 3’IVT PLUS Express kit (900720, Affymetrix, Thermo Fisher Scientific, USA), yielding a fragmented biotin-labeled cRNA library. After adding internal probe array controls to the target samples for signal normalization, the mixtures were hybridized with GeneChip *Drosophila* 2.0 (Affymetrix, Thermo Fisher Scientific, USA), and then washed and stained with a Hybridization, Wash and Stain Kit (900720, Affymetrix, Thermo Fisher Scientific, USA). All procedures were performed according to the manufacturer’s protocol. After scanning fluorescent signals from cRNA-chip matrix heteroduplexes, data transformation was conducted with the RMA algorithm and quality control was performed using GeneChip Expression Console Software 1.4 (Affymetrix, Thermo Fisher Scientific, USA). Eventually, lists of up- and downregulated genes in knockdown flies compared to control flies were obtained, assessing more than 18,500 transcripts with TAC4.0 (Affymetrix, Thermo Fisher Scientific, USA). Functional enrichment analysis of these data files was performed with g:Profiler [39]. For analysis, only the genes with more than a two-fold change in expression (False Discovery Rate (FDR) < 0.05) were considered and the gene groups divided by the Gene Ontology (GO) biological process were studied (adjusted *p*-value < 0.05). 

### 2.12. Results Processing

Statistical analysis was performed using KyPlot 5.0 software. All samples were tested for normality with the Shapiro–Wilk test. If the distribution was normal, we used parametric tests: The Student t-test for a comparison of two samples and Tukey–Kramer test for multiple comparisons (three or more samples). For normally distributed samples, data were presented as histograms (mean ± 95% confidence interval). For other distribution types, nonparametric tests were used. For a comparison of two samples, the Kruskal–Wallis test was applied, whereas for multiple comparisons (three or more samples), the Steel–Dwass test was performed. For samples where the Shapiro–Wilk test suggested a non-normal distribution, data were presented as box-and-whisker plots. In most cases where there were several technical replicates, we did not average them, but put all the values in one sample, assuming that each value is an independent representative of an entire assembly of a studied parameter (an exception was ROS analysis, described in 2.9).

## 3. Results

### 3.1. sws is Expressed in the Central and Peripheral Nervous System in Drosophila Larva and Imago

Previous studies have shown that the SWS protein is expressed in the interface glia at the neuropil–cortex border, fenestrated and satellite glia in the lamina, medulla neuropile glia at the medulla cortex–neuropil interface, and most of the neurons [10]. Later, it was shown that *sws* is necessary in SPG and ensheathing glia [11] and its dysfunction leads to neuronal and glial death. It was also initially shown that glial hyperwrapping exists in *sws* mutants [9]. In our previous work, we found that *sws* was mainly detected on the surface of the ventral nerve cord, in abdominal nerves, and presynaptically at the neuro-muscular junctions of PNS of the 3rd instar larvae [40].

**Figure 2:**
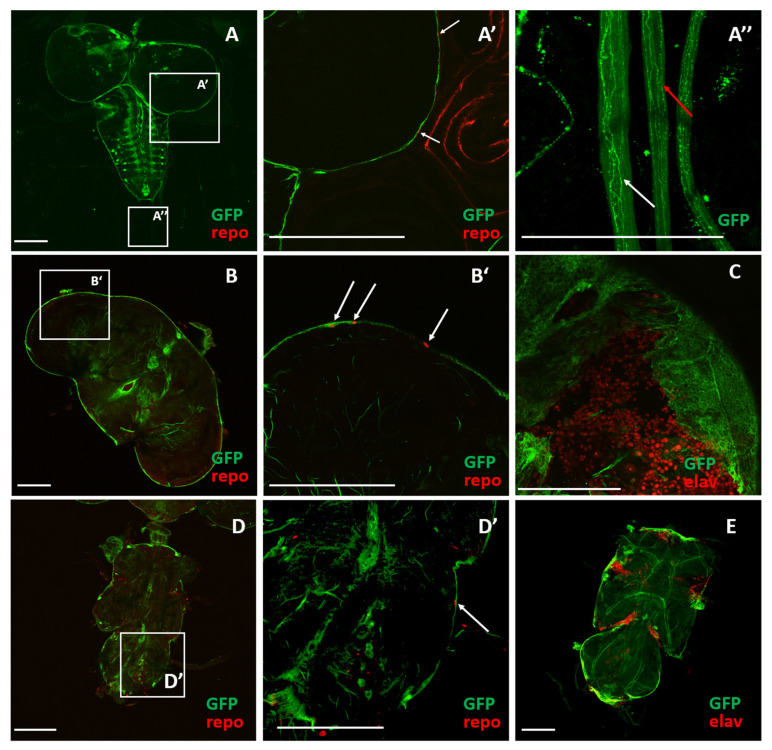
Expression pattern of the *sws* gene in the *Drosophila* nervous system. (**A–E**) Visualization of the *sws* expression pattern in the transgenic *Drosophila melanogaster* larval and imago nervous system (*sws-GAL4;UAS-CD8-GFP*) revealed by confocal microscopy. Scale bar: 100 μm. Green: GFP embedded in membranes of cells with an active *sws* promoter. Red: (**A–A’, B–B’, D–D’**) glial cell nuclei in the brain and ventral nerve cord, labeled with anti-repo antibodies (marked with white arrows); (**C, E**) neuronal nuclei labeled with anti-Elav antibodies. (**A–A’**) Brain of 3rd instar larva; (**B–B’, C**) brain of 5-day-old imago; (**D–D’, E**) ventral nerve cord of 5-day-old imago. (**A, B, D**) Whole view; (**A’, B’, D’**) magnification; (**A’’**) abdominal nerves of 3rd instar larva; white arrows show the GFP expression in the abdominal nerves and on their surfaces.

In this study, we were able to visualize cells with an active *sws* promoter in the tissues of *sws-GAL4;UAS-CD8-GFP* 3rd instar larvae and imagoes at the 5th and 30th day of life. We found strong signal in the outer layers of the brain in all examined developmental stages: Plausibly PG and SPG, overlapping with the repo-positive nuclei on the brain surface (Figure 2A–A’, B–B’, D–D’). In addition, there was a weak expression inside the brain and ventral nerve cord (Figure 2A,B). The pictures (Figure 2C,E) show that neuronal nuclei do not overlap with the GFP signal in the brain cortex and ventral nerve cord, suggesting that the *sws* expression in the neuronal cell bodies and CG is extremely low or absent. In the larval ventral nerve cord, there were several pronounced *sws* expressing regions corresponding to the metameric structure of thoracic and abdominal ganglia and respective nerves (Figure 2A–A’’). 

### 3.2. The sws Gene is Important for the Structural Maintenance of SPG in CNS

**Figure 3:**
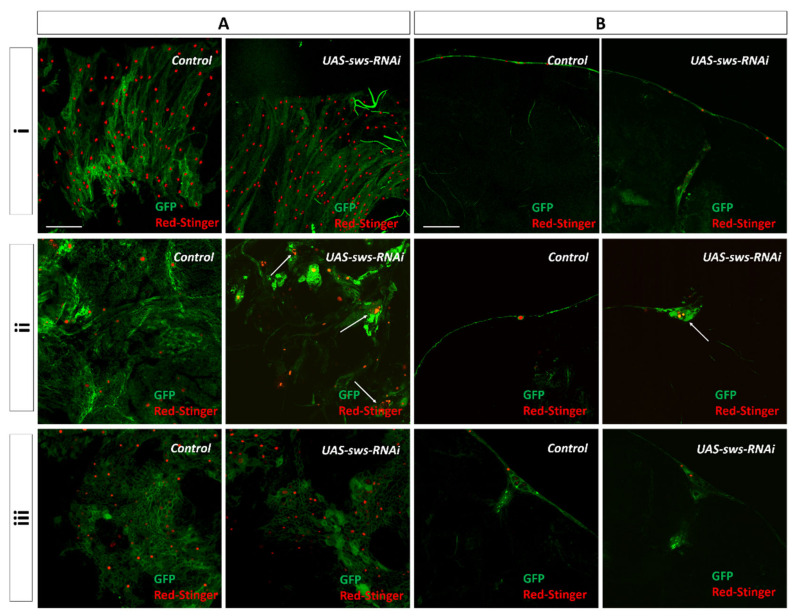
Glia morphology under *sws* knockdown in the central nervous system (CNS). (**A**–**B**) Visualization of glial cells in brains of 30-day-old flies marked with membrane GFP (green, *UAS-CD8-GFP*) and nuclear red fluorescent protein, RFP (red, *UAS-RedStinger*) expression in (**i**) perineurial glia (PG) (*NPP-GAL4*), (**ii**) SPG (*NPS-GAL4*), (**iii**) cortex glia (CG) (*NPC-GAL4*) in control and knockdown (KD) (*UAS-sws-RNAi*) flies. (**A**) Confocal image of glial cells on the brain surface, and (**B**) cross-sectional confocal image of the brain. White arrows indicate fragmented glia nuclei. Scale bar: 50 μm.

Since the *sws* promoter is highly active in the outer layer of the brain and, according to the data on the role of *sws* in SPG [11], we were curious to know whether the *sws* gene is important for *Drosophila* BBB, which is known to be mostly dependent on the SPG function [15,31,41]. This type of glia lies between PG and CG and these three types of glia interact with each other [18]. We did not reveal any signal in the cortex of the brain (Figure 2C). We suspected that *sws* could also be expressed in PG because we revealed signals near the red repo-positive nuclei of different sizes and forms, suggesting that the *sws* promoter is active in both SPG and PG (Figure 2A–A’, B–B’). Moreover, it has not previously been studied whether *sws* has any functional role in PG, even though it was shown that SWS protein was found in the lamina fenestrated glia (a subtype of PG) [10]. Therefore, we knocked down *sws* using *RNAi* and various glia-specific drivers in PG (*NPP-GAL4;UAS-sws-RNAi*), SPG (*NPS-GAL4;UAS-sws-RNAi*), and CG (*NPC-GAL4;UAS-sws-RNAi*). For the latter, the driver designated as *NPC-GAL4* is also expressed in WG [19,42], so it allowed us to analyze the morphology of CG in the CNS and WG in the PNS independently. In order to dissect the role of *sws* in PG, SPG, and CG, we analyzed the morphology of these glia subtypes in KDs at different stages of *Drosophila* development. We also used additional transgenes to visualize the cell membranes (*UAS-CD8-GFP*, shown in green, Figure 3) and the nuclei (*UAS-RedStinger*, shown in red, Figure 3).

Downregulation of *sws* in PG and CG using the *NPP-GAL4* and *NPC-GAL4* drivers did not cause the apparent morphological changes.

Additionally, we did not notice any morphological alterations in the larval ganglia under *sws* KD in SPG. However, in the adult flies, we observed pronounced abnormalities in the SPG layer, which progressed with age. In 5-day-old imago, the SPG layer became discontinuous, gaps were found on the surface of the brain, and the structure of the glia was changed. In 30-day-old animals, the loss of SPG integrity was enhanced: Glial cells on the surface of the brain were grouped together, forming conglomerates with extensive gaps between them (Figure 3iiA,B, arrow; a more detailed structure of SPG is presented in Supplementary Figure S2). In addition, the visualization of DsRed in SPG nuclei shows their fragmentation (Figure 3A, white arrows), which is typical for apoptotic cells [43,44]. 

### 3.3. The sws Gene is Important for the Structural Maintenance of SPG and WG in PNS

**Figure 4:**
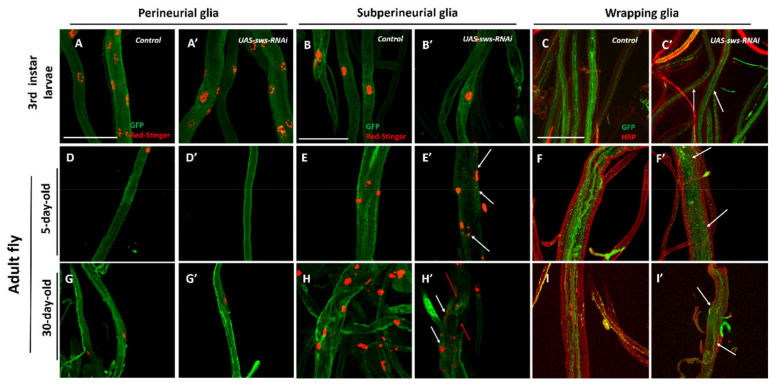
Glia morphology under *sws* knockdown in PNS. (**A**–**I’**) Morphology of glial cells in PNS (abdominal proximal nerves): (**A**–**A**’, **D**–**D’**, **G**–**G’**) PG (*NPP-GAL4*); (**B**–**B’**, **E**–**E’**, **H**–**H’**) SPG (*NPS-GAL4*); and (**C**–**C’**, **F**–**F’**, **I**–**I’**) WG (*NPC-GAL4*) in 3rd instar larvae (**A**–**A’**, **B**–**B’**, **C**–**C’**), and 5-day-old (**D**–**D’**, **E**–**E’**, **F**–**F’**) and 30-day-old (**G**–**G’**, **H**–**H’**, **I**–**I’**) adult flies of control or KD (*UAS-sws-RNAi*) genotype. Green: GFP in membranes (*UAS-CD8-GFP*). Red: (**A**–**B’**, **D**–**E’**, **G**–**H’**) RFP expression in nuclei (*UAS-RedStinger*) or (**C**–**C’**, **F**–**F’**, **I**–**I’**) red anti-HRP antibody staining of axons. (**E’**, **H’**) White arrows show an increased number of SPG nuclei, and red arrows show a loss of the SPG layer. (**C’**, **F’**, **I’**) White arrows show a loss of WG. Scale bar: 50 μm.

Abdominal nerves, which are part of the PNS, are present in *Drosophila melanogaster* from the early stages of larval development [45]. In the course of the fly life cycle, abdominal nerves are modified during metamorphosis. Therefore, five posterior nerve pairs (A4–A8) merge to form the terminal nerve [46]. In this work, we analyzed three types of PNS glia (PG, SPG, WG) in the abdominal nerves of 3rd instar larva (nerves A4–A8, Figure 4A–C’), 5-day-old flies (terminal nerve, Figure 4D–F’), and 30-day-old adult flies (terminal nerve, Figure 4G–I’). 

We did not find any obvious morphological alterations in the 3rd instar larva abdominal nerves in the case of *sws* KD in PG or SPG (Figure 4A,B). However, upon *sws* KD in SPG in 5-day-old imago, the SPG layer of the terminal nerve lost its integrity and this phenotype was enhanced with age. In addition, we observed the fragmentation of SPG nuclei in PNS, similar to what we saw in CNS (Figure 4E’,H’, white arrows). 

The KD of *sws* in WG resulted in morphological abnormalities in 3rd instar larva (Figure 4C–C’, white arrows). This phenotype was also found in 5-day-old imagoes, where WG lost its regular structure (Figure 4F’, white arrows). In 30-day-old imagoes, there was an almost complete loss of the WG layer (Figure 4I’, white arrows).

### 3.4. Knockdown of sws in Glia Leads to Degeneration in CNS

The observed morphological phenotypes could be due to the *sws*-deficit-induced degeneration that was previously described [9,10,11]. We quantitatively assessed this in two ways: First, we counted the nuclei number of each cell type in the CNS, and second, we measured the total hole area in the brain neuropile. 

**Figure 5:**
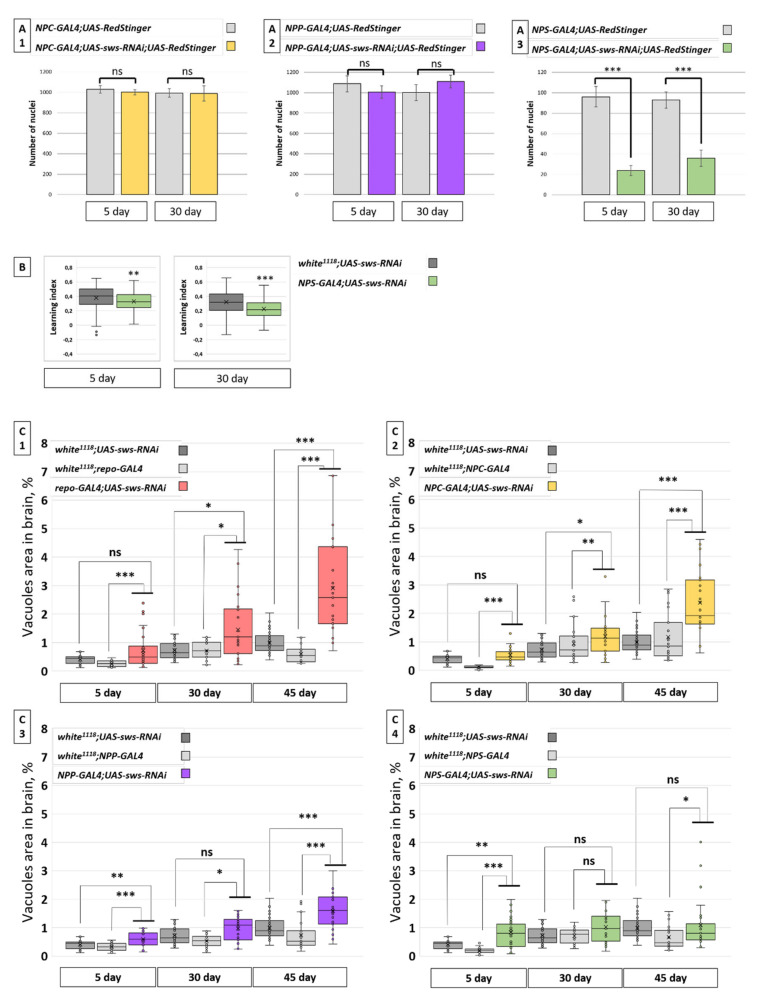
Analysis of degeneration in the fly brains. (**A**) Number of CG (**A1**), PG (**A2**), and SPG (**A3**) nuclei in fly brains with KD of *sws* in corresponding glial cells (*UAS-sws-RNAi*). Student *t*-test, mean ± 95%CI, *N* = 25. (**B**) The learning index for flies with *sws* KD in SPG and controls (F1 males from crossing *w^1118^* female and *UAS-sws-RNAi* male, gray boxes). Kruskal–Wallis test, ** *p* < 0.01, *** *p* < 0.001, *N* = 91. (**C**) Box-and-whisker plots for the total hole area in fly brains with *sws* KD in all glia types (**C1**), CG (**C2**), PG (**C3**), and SPG (**C4**). Controls are F1 males obtained from crossing a *w^1118^* female and *UAS-sws-RNAi* male (dark gray boxes) and F1 males obtained from crossing *w^1118^* females and one of each of the four *GAL4* males used (light gray boxes). Steel–Dwass test, * *p* < 0.05, ** *p* < 0.01, *** *p* < 0.001; ns, no significant difference (*p* > 0.05), *N* = 27.

We found that *sws* KD had no influence on the number of CG glia nuclei in a fly’s brain (Figure 5A1). Moreover, the PG nuclei numbers did not significantly change when *sws* was downregulated in this cell type (Figure 5A2). In contrast, we observed a severe reduction of the number of SPG glia nuclei, even at the 5th day of the imago’s life. This number, however, did not seem to reduce further upon aging (Figure 5A3). 

Considering that SPG is the main component of BBB, next, we addressed the question of whether the brain function is affected in flies with SPG *sws* KD. Through the classical Pavlovian olfactory assay, we were able to assess the *Drosophila* learning index. We found that *sws* KD in SPG reduces the index in groups of 5- and 30-day-old flies (Figure 5B).

Next, we analyzed the total hole area in fly brains with glia-specific *sws* KD to assess whether alterations of glia have an impact on CNS axon survival and could potentially cause a “swiss cheese” phenotype (Figure 5C) [9]. Firstly, we analyzed the phenotype under panglial *sws* KD in *repo-GAL4;UAS-sws-RNAi* flies. We noticed a slight age-dependent increase of the total neuropile degeneration level (Figure 5C1). Surprisingly, a similar pattern was observed in *NPC-GAL4;UAS-sws-RNAi* (CG *sws* KD, Figure 5C2) and *NPP-GAL4;UAS-sws-RNAi* (PG *sws* KD, Figure 5C3) flies. A quite different picture was revealed for SPG *sws* KD: There was only an increase in the vacuole area compared to controls in 5-day-old flies. These data demonstrate that the analyzed brain degeneration index only slightly increased in flies with *sws* KD in the examined glia types. Therefore, we propose that the downregulation of *sws* expression in glia is not critical for whole brain integrity and neuronal survival (Supplementary Table S1).

### 3.5. Glial Knockdown of sws is Associated with Impaired Behavior

To assess the functional consequences of the morphological alterations observed, we analyzed some common neurobiological traits, namely, the organism survival (lifespan) and behavior. The lifespan is often used as a combined biological measure of the functional state of flies [47]. We did not reveal any lifespan reduction in any of the four glia-specific *sws* KDs, suggesting that *sws* expression decline in glia is not fatal for flies (Supplementary Figure S3).

**Figure 6:**
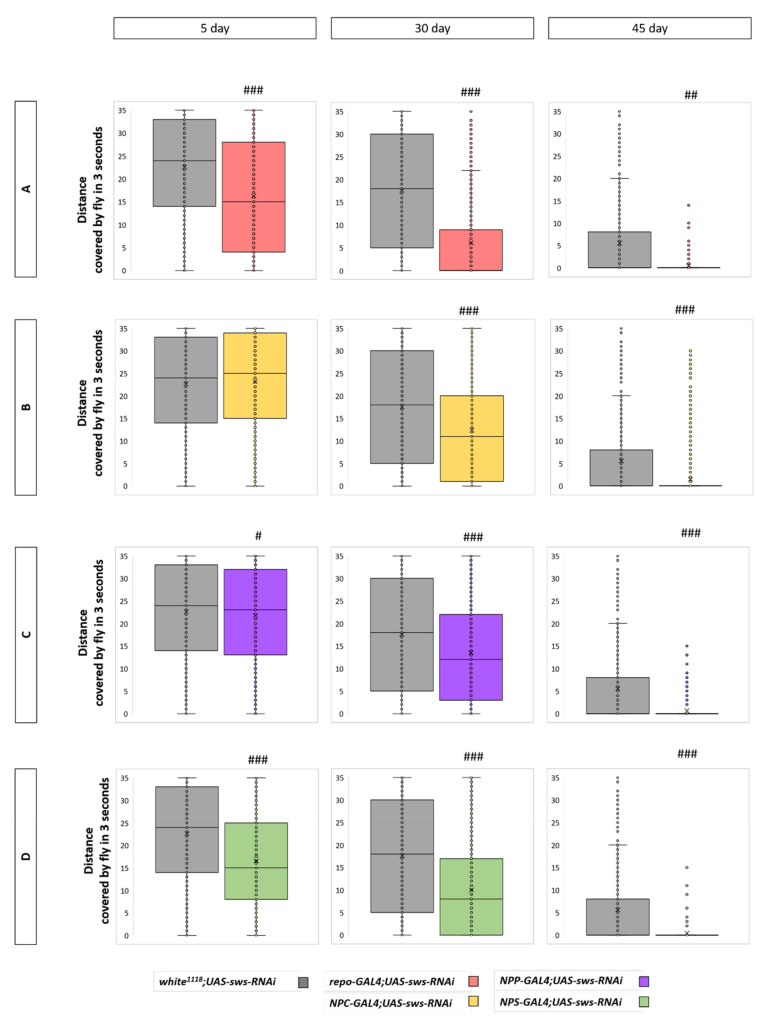
Locomotor activity analysis. (**A**–**D**) Distance covered by flies for 3 s after flipping in the RING assay for flies with *sws* KD in all types of glia (**A**), CG + WG (**B**), PG (**C**), SPG (**D**), and controls (F1 males obtained from crossing a *w^1118^* female and *UAS-sws-RNAi* male) at different ages. Kruskal–Wallis test, # *p* < 0.05, ## *p* < 0.01, ### *p* < 0.001, *N* > 1200.

It was previously shown that, in a fast phototaxis assay, the locomotion activity of flies with the *Gli-GAL4;sws^GD3277^* genotype is reduced, where *sws* is knocked down in SPG [21]. When trying to find out whether the morphological defects observed have behavioral consequences, we analyzed locomotor activity in a negative geotaxis test (Figure 6). We found that in all four *sws* KDs, the locomotor index was reduced in 30- and 45-day-old flies, suggesting that *sws* is important in PG, SPG, and CG + WG for locomotor performance. Moreover, in SPG glia *sws* KD, as well as in panglial *sws* KD, the index was lower compared to controls, even in 5-day-old flies, which is in accordance with our morphology and neurodegeneration data for these genotypes.

### 3.6. Whole-Organism Transcriptome Analysis of Flies with sws KD

Being a regulatory subunit of protein kinase A [7,8], the SWS protein can influence the gene expression profile, thus making it possible to reveal the changes at the transcriptomic level in response to changes in *sws* expression levels. Moreover, the impairment of functions that are under the control of the *sws* gene can lead to intracellular physiology fluctuations and intercellular communication deviations; therefore, having an influence not only on cells with *sws* KD, but also on the whole organism (or at least on the nervous tissue that in its turn is an orchestrator of multiple processes). In addition, the observed morphology phenotypes may induce some other organism reactions, such as inflammation. Taking these statements into account, we tried to assess the organism response to *sws* KD in SPG and CG + WG. Therefore, whole-fly transcriptome analysis was performed. We studied changes of transcript levels in adult 30-day-old SPG and CG + WG *sws* KDs compared to *CantonS* control flies. In the case of SPG *sws* KD, we found 856 genes to be upregulated. These genes control the organism and cell response to infection, immunity, proteolysis, and metabolism of carboxylic acids and glutathione (Supplementary Figures S4 and S5). Among the 541 genes that were downregulated, functional enrichment analysis revealed reproductive processes and protein metabolism to be overrepresented (Supplementary Figures S6 and S7). As for CG + WG *sws* KD, we showed 1026 upregulated genes. They control the metabolism of various molecules, including carboxylic acids, lipids, amino acids, carbohydrates, xenobiotics, flavonoids, glucuronate, and glutathione (Supplementary Figures S8 and S9). Among the 691 downregulated genes, there were several statistically significant overrepresented processes regulating organism reproduction (Supplementary Figure S10 and S11). 

### 3.7. Glial Knockdown of sws is Associated with Increased ROS Levels

**Figure 7:**
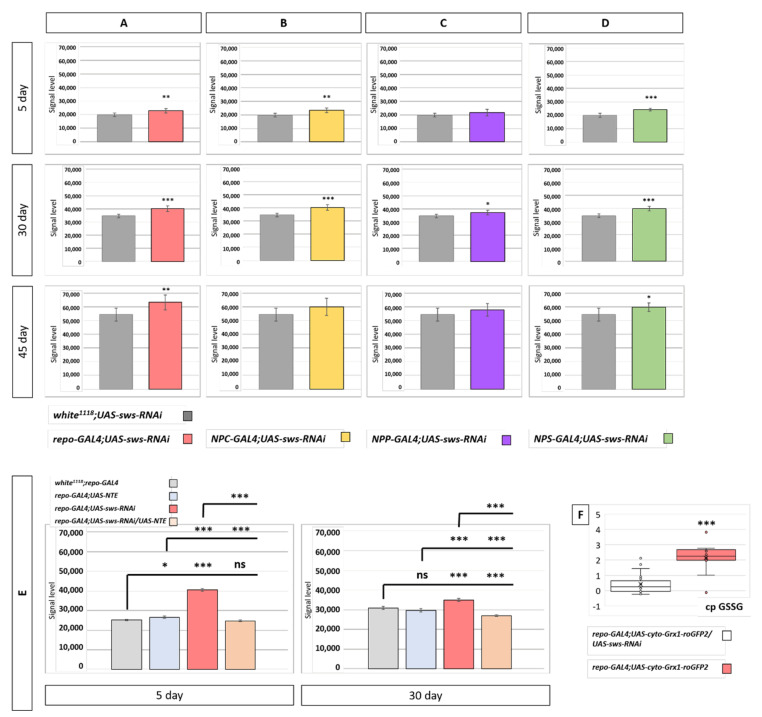
Reactive oxygen species analysis. (**A**–**D**) Fluorescent signal level corresponding to the total reactive oxygen species (ROS) concentration in brain samples of flies with *sws* KD in all glia (**A**), CG (**B**), PG (**C**), SPG (**D**), and controls (F1 males obtained from crossing a *w^1118^* female and *UAS-sws-RNAi* male) at different ages. Student *t*-test, mean ± 95% CI is shown, *N* = 7. (**E**) Fluorescent signal level corresponding to the total ROS concentration in brain samples of *repo-GAL4* flies with human *NTE* overexpression (*UAS-NTE*), *sws* KD in all types of glia (*UAS-sws-RNAi*), and rescue of *sws* loss (*UAS-NTE/UAS-sws-RNAi*). Controls are F1 males from crossing a *w^1118^* female and *repo-GAL4* male. Tukey–Kramer test, mean ± 95%CI is shown. * *p* < 0.05; ** *p* < 0.01; *** *p* < 0.001; ns, no significant difference (*p* > 0.05) N = 7. ( **F**) Relative level of oxidized glutathione in the glia cytoplasm of panglial *sws* KD (*repo-GAL4;UAS-cyto-Grx1-roGFP2/UAS-sws-RNAi*, red box) and controls (*repo-GAL4;UAS-cyto-Grx1-roGFP2*, gray box) at the 30th day of life. Kruskal–Wallis test, *** *p* < 0.001, *N* = 12. OY axis, relative ratio of sensor fluorescence.

It is known that neurodegenerative phenotypes are often accompanied by ROS acceleration [48]. Therefore, we performed brain ROS level analysis using an H2DCF-DA probe in whole-head lysates (Figure 7A–D). We found slight ROS acceleration in all glia *sws* KD flies (*repo-GAL4;UAS-sws-RNAi*) and in SPG *sws* KD flies at 5, 30, and 45 days of life. Additionally, there was ROS increase in 5- and 30-day-old CG *sws* KD. To confirm that the observed ROS acceleration is induced by *sws* downregulation, we performed a rescue experiment. For this, we expressed a human *sws* orthologue—*NTE*—in a *repo-GAL4;UAS-sws-RNAi* background, revealing ROS level reduction to normal values (at the 5th day of life) or even less than the control level (at the 30th day of life, Figure 5E). Having revealed that under SPG and CG + WG *sws* KD, glutathione metabolism genes are upregulated, we decided to analyze whether the observed ROS acceleration is at least partially utilized by the glutathione antioxidant system. Therefore, we analyzed the relative oxidized glutathione level in brains of panglial *sws* KD (*repo-GAL4;UAS-sws-RNAi*) flies and found an elevation of it compared to the control (Figure 7F). 

## 4. Discussion

Glial cells are known to be essential elements of the nervous system that are critical for the development and maintenance of neurons, including their protection from changing environments [49,50,51,52,53]. *Drosophila melanogaster* is a great model for investigating developmental, morphological, and functional aspects of glia biology [18,54,55,56]. Among multiple neuron–glia interactions, one of the most vital is hemolymph-neuron protective isolation performed by glia in the fruit fly [13,57,58]. Recent studies have identified numerous genes that are associated with specific glia functioning in *Drosophila* [21,29,30,31]. Here, we show that the *sws* gene is important for glia maintenance in *Drosophila melanogaster*. We found that *sws* is expressed not only in imago’s neuropil, but also in brain surface and nerves (where BBB/BNB glia are located) at the larval stage of development (when BBB/BNB glia grow [59]), as well as at the imago stage. Therefore, we propose that the *sws* function is important for, at least, the late development and maintenance of BBB/BNB glia. 

We identified that both SPG and WG require a normal *sws* expression for structural stability. The former glia type is known to be a major constituent of insect BBB/BNB [15,16]. The latter type of glia serves as non-myelinating cells that ensheath axons in peripheral nerves to provide both proper conductance and nerve integrity [15,28]. We suppose that the observed glia impairment may be associated with the *sws* function. It has been well-demonstrated that lipid metabolism in glia is crucial for stress reaction under different conditions, primarily associated with oxidative stress [60,61,62]. Being a phospholipase [10], SWS plays an important role, not only for lipid metabolism, but also for stress regulation, thus providing cell metabolism and survival [63]. This could explain why the downregulation of *sws* in the case of KD results in ROS elevation in the brain, suggesting that a loss of the glia defense function occurs. 

To check whether this hypothesis is true, we applied RNA-interference-induced KD of *sws* in those cell types that could influence *Drosophila* BBB and BNB. Among the studied glia types, it was shown that only SPG demands *sws* expression for a normal brain condition. The KD of *sws* in SPG led to membranous structures and vacuole formation in the lamina cortex [11]. However, the SPG-specific function of *sws* was not shown. We demonstrated that a lack of *sws* expression in SPG disrupted the layer of these cells in 5-day-old flies and the SPG nuclei number was reduced. This phenotype progressed to the 30th day of adult life. In addition, not only the structure of the cell layer, but also numerous fragmented nuclei, accumulated in residual SPG “islets” and were typical for apoptotic cells [43,44]. These abnormalities were observed in both CNS and PNS. The phenotype was accompanied by a locomotor activity decline in the negative geotaxis test, supporting previous data about defects in a fast phototaxis assay for *Gli-GAL4;sws^GD3277^* flies (another variant of SPG *sws* KD) [11]. It was demonstrated that in case of human neurodegenerative disorders with even mild memory disturbances, BBB leaking occurs [64,65,66]. Hence, proposing an alteration of BBB integrity in our flies, we analyzed the cognitive function of SPG *sws* KDs. We found a learning (a.k.a. immediate memory) deficit in examined flies. The general morphological state of the brain seemed to be normal, even though there was a slight neuropile degeneration index increase. However, the brain ROS level was accelerated in SPG *sws* KD flies, suggesting that the brain function was altered under an induced condition. Despite the observed pathological phenotypes, the longevity of KD flies did not change. Nevertheless, these data suggest that the main cell type of BBB—SPG—demands a normal *sws* expression for maintenance and function. The KD of *sws* in SPG leads to dramatic morphological alterations, oxidative stress, and behavior deficits.

As for PG *sws* KD, we did not find any morphological abnormalities. However, we found a few more vacuoles in the brains of these flies; however, we still propose that this could be part of normal variation because of the small effect size. The small increase of the brain ROS level in 30-day-old KD had quite low statistical significance (*p* = 0.018). However, we found a locomotor activity decline at day 30 and 45. Therefore, the potential role of *sws* in PG cells has to be studied in more detail. On the one hand, we were not able to show whether the *sws* promoter is active in both PG and SPG layers, or only in one of them. On the other hand, there is no doubt that *sws* is necessary, at least in SPG. Our data suggest that the locomotor activity decline observed in PG *sws* KD could be an argument to further investigate the role of *sws* in this glia subtype.

We observed no visible activity of the *sws* promoter in CG. Additionally, we found no morphology and nuclei number changes under CG *sws* KD. Surprisingly, there was increased vacuole formation in the brain neuropile and an accelerated ROS level. This was accompanied by a locomotor activity decline. While the brain phenotype is puzzling for us, the locomotor phenotype could be associated with WG, because we used the same driver for both cell types (*NPC-GAL4*). In WG, we found morphological abnormalities in 3rd instar larvae, and the phenotype progressed in imago, resulting in the complete loss of WG in peripheral nerves to the 30th day.

To assess organism changes under SPG or CG + WG *sws* KD in 30-day-old imagoes, we analyzed the transcriptome of whole flies. This let us roughly analyze general changes in organisms compared to the control with normal *sws* expression (*CantonS*). Hundreds of genes were up- or downregulated under the KD condition. In the case of SPG *sws* KD, there were genes controlling the immunity and defense reaction. This could indicate consequences of BBB impairment. As for CG + WG *sws* KD, there were various genes controlling metabolism. Considering the fact that these two types of glia are tightly associated with neurons and supply them with nutrients, KD could alter respective neuron–glia interactions. We found that in both SPG and CG + WG *sws* KDs, glutathione metabolism genes were upregulated. Glutathione is known to be the major natural endogenous antioxidant in a wide variety of species, including fruit flies. We therefore performed an analysis of the relative oxidized glutathione level in glia and revealed an elevation of this parameter in 30-day-old fly brains with panglial *sws* KD. This confirms our results about ROS acceleration in fly brains of several genotypes. In addition, organism reproduction genes were downregulated in both KDs. This could be due to an organism stress reaction, which implies activation of the defense response and organism survival.

It was previously documented that *sws* dysfunction results in neuronal and glial cell death [9,10,11]. This leads to locomotor deficits due to both neuronal and glial dysfunction. For instance, neuromuscular junction alterations are accompanied with by an active zone decline and postsynaptic reaction to this deterioration due to the neuronal *sws* KD in *Drosophila* [40]. The human *sws* orthologue is associated with several syndromes of the spasticity—ataxia continuum caused by neurodegeneration in the spinal cord, cerebellum, and/or pituitary gland [67]. The murine *sws* orthologue is necessary for non-myelinating Schwann cells to ensheath axons in Remak fibers [68]. In the fruit fly, a similar function is performed by WG, which we demonstrated to be dependent on *sws* for peripheral nerve structural maintenance and locomotor activity. Furthermore, SPG glia could not properly ensheath ganglia and nerves under *sws* KD. Being normal in larvae, they are severely disrupted in young imagoes. Although we did not directly show that the two mentioned cell types die in the case of *sws* KD, the gene is crucial in these cells not only for proper morphology maintenance, but also for the normal functional state of the organism. 

## 5. Conclusions

Taken together, we have shown that normal *sws* expression is important for the regular structure of SPG in adults and WG in both 3rd instar larvae and adults. Under *sws* KD, these cell types are responsible for the locomotor activity decline. Moreover, the SPG KD phenotype is associated with an elevated ROS level in the brain and learning deficiency. However, the mechanisms of neurodegeneration under *sws* knockdown, as well as an evidence for the role of *sws* in PG and CG, remain unknown. 

## Data Availability

The data presented in this study are available in the article and Supplementary Materials.

## Supplementary Materials

The following are available online at https://www.mdpi.com/2073-4409/10/3/529/s1: Supplementary Table S1. Neuropile degeneration index (total vacuole area in the brain neuropile) in flies with different genotypes and three ages; Supplementary Figure S1. Automated analysis of the SPG nuclei in ImageJ; Supplementary Figure S2. Subperineurial glia morphology under *sws* knockdown in the CNS; Supplementary Figure S3. Longevity assay; Supplementary Figure S4. Processes under the control of upregulated genes in 30-day-old SPG *sws* KD males compared to *CantonS* control and respective FDR-adjusted p-values of functional enrichment analysis in g:Profiler software; Supplementary Figure S5. Processes under the control of upregulated genes in 30-day-old SPG *sws* KD males compared to *CantonS* control and respective query gene number from functional enrichment analysis in g:Profiler software; Supplementary Figure S6. Processes under the control of downregulated genes in 30-day-old SPG *sws* KD males compared to *CantonS* control and respective FDR-adjusted p-values of functional enrichment analysis in g:Profiler software; Supplementary Figure S7. Processes under the control of downregulated genes in 30-day-old SPG *sws* KD males compared to *CantonS* control and respective query gene number from functional enrichment analysis in g:Profiler software; Supplementary Figure S8. Processes under the control of upregulated genes in 30-day-old CG + WG *sws* KD males compared to *CantonS* control and respective FDR-adjusted p-values of functional enrichment analysis in g:Profiler software; Supplementary Figure S9. Processes under the control of upregulated genes in 30-day-old CG + WG *sws* KD males compared to *CantonS* control and the respective query gene number from functional enrichment analysis in g:Profiler software; Supplementary Figure S10. Processes under the control of downregulated genes in 30-day-old CG + WG *sws* KD males compared to *CantonS* control and respective FDR-adjusted p-values of functional enrichment analysis in g:Profiler software; Supplementary Figure S11. Processes under the control of downregulated genes in 30-day-old CG + WG *sws* KD males compared to *CantonS* control and the respective query gene number from functional enrichment analysis in g:Profiler software.
