# Supplementary material for: Morpho-Functional Consequences of Swiss Cheese Knockdown in Glia of Drosophila melanogaster
Source: Cells. 2021 Mar 2;10(3):529. doi: 10.3390/cells10030529 (PMC7998100; doi:10.3390/cells10030529)
Supplement: Supplementary file 1 [file cells-10-00529-s001.pdf]

Genotype	Brain Vacuole Area (Median), %		
	5 day	30 day	45 day
w^{1118}; UAS-sws-RNAi	0.45	0.64	0.89
w^{1118}; repo-GAL4	0.26	0.71	0.54
repo-GAL4; UAS-sws-RNAi	0.49	1.20	2.58
^{w1118}; NPP-GAL4	0.33	0.54	0.53
NPP-GAL4; UAS-sws-RNAi	0.59	1.09	1.60
w^{1118}; NPS-GAL4	0.20	0.78	0.48
NPS-GAL4; UAS-sws-RNAi	0.81	0.97	0.80
w^{1118}; NPC-GAL4	0.19	0.72	0.85
NPC-GAL4; UAS-sws-RNAi	0.47	1.13	1.92

Table S1. The neuropile degeneration index (total vacuole area in brain neuropile) in flies with different genotypes and three ages.

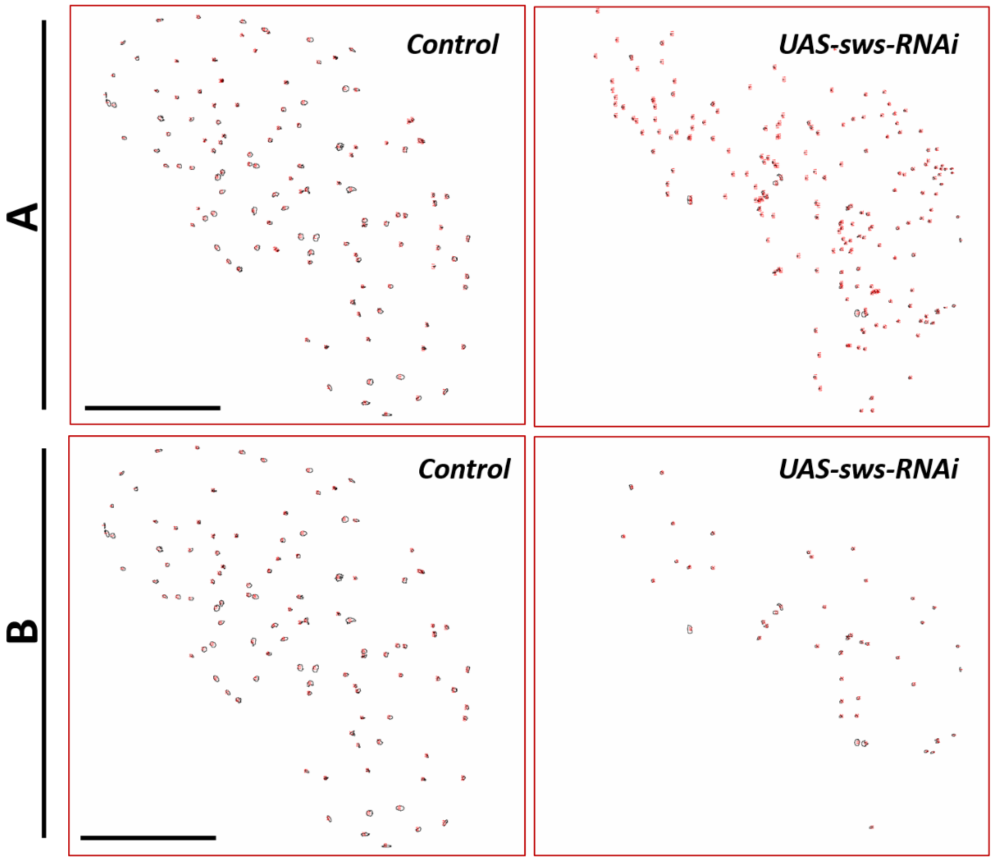

Figure S1. Automated analysis of the SPG nuclei in ImageJ. **(A)** the minimum size of nuclei is 5 μm^2 in the Control and *sws* KD (*UAS-sws-RNAi*). **(B)** the minimum size of normal nuclei is 10 μm^2 in the Control and *sws* KD (*UAS-sws-RNAi*). Scale bar: 25 μm.

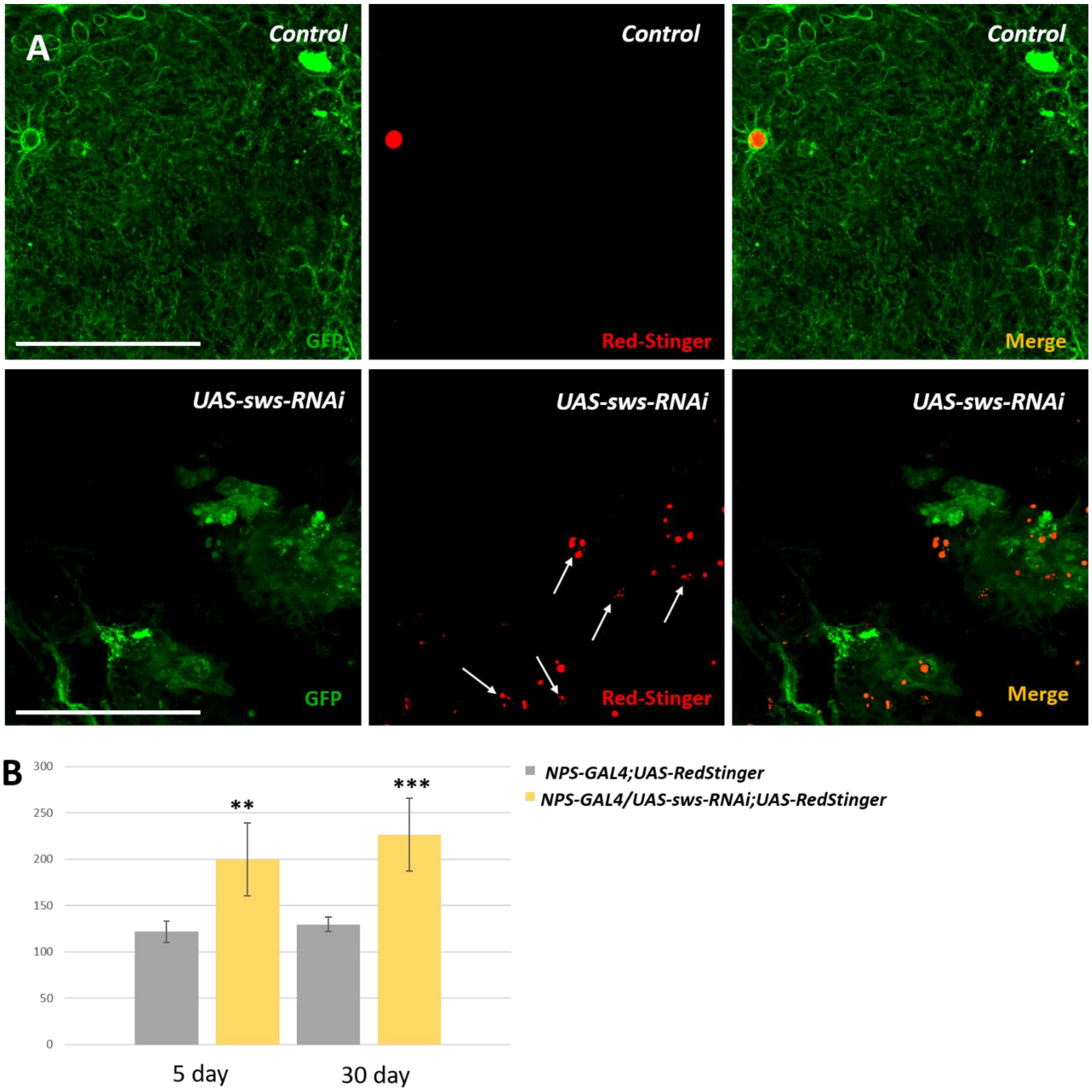

Figure S2. Subperineurial glia morphology under *sws* knockdown in the CNS. (**A**) Visualisation of subperineurial glia in brains of 30-day-old flies marked with membrane GFP (green, UAS-CD8-GFP) and nuclear RFP (red, UAS-RedStinger) expression in SPG (NPS-GAL4) in control and knockdown (UAS-sws-RNAi) flies. White arrows indicate Red-Stinger-positive fragmented nuclei of glia. Scale bar; 25 μm. (**B**) Analysis number of SPG nuclei (5 μm^2 size) in fly brains with *sws* KD(UAS-sws-RNAi). Student t-test, mean ± 95% CI, n = 25.

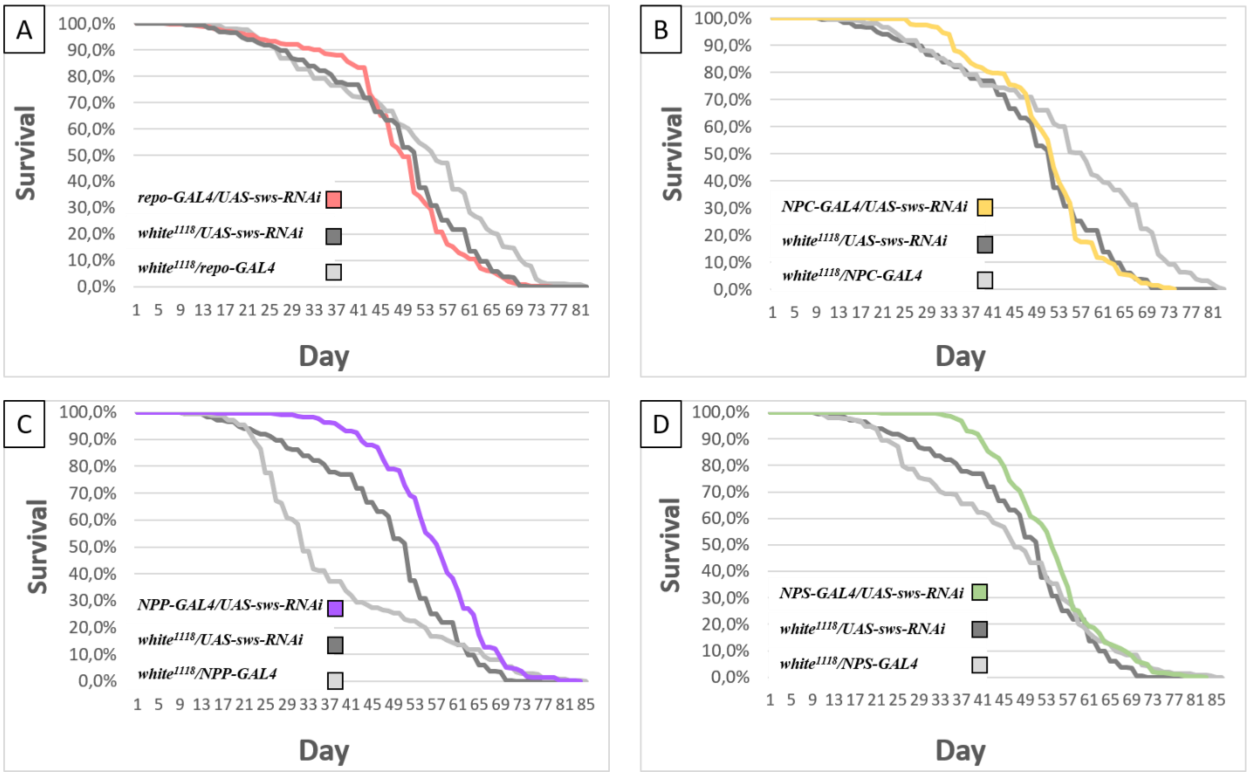

Figure S3. Longevity assay. (A-D) Survival curves for flies with *sws* KD in all glia (**A**), CG+WG (**B**), PG (**C**) and SPG (**D**). Controls are F1 males from crossing w^{1118} female and *UAS-sws-RNAi* male (dark grey lines) and F1 males from crossing w^{1118} females and one of each of the four used *GAL4* males (light grey lines).

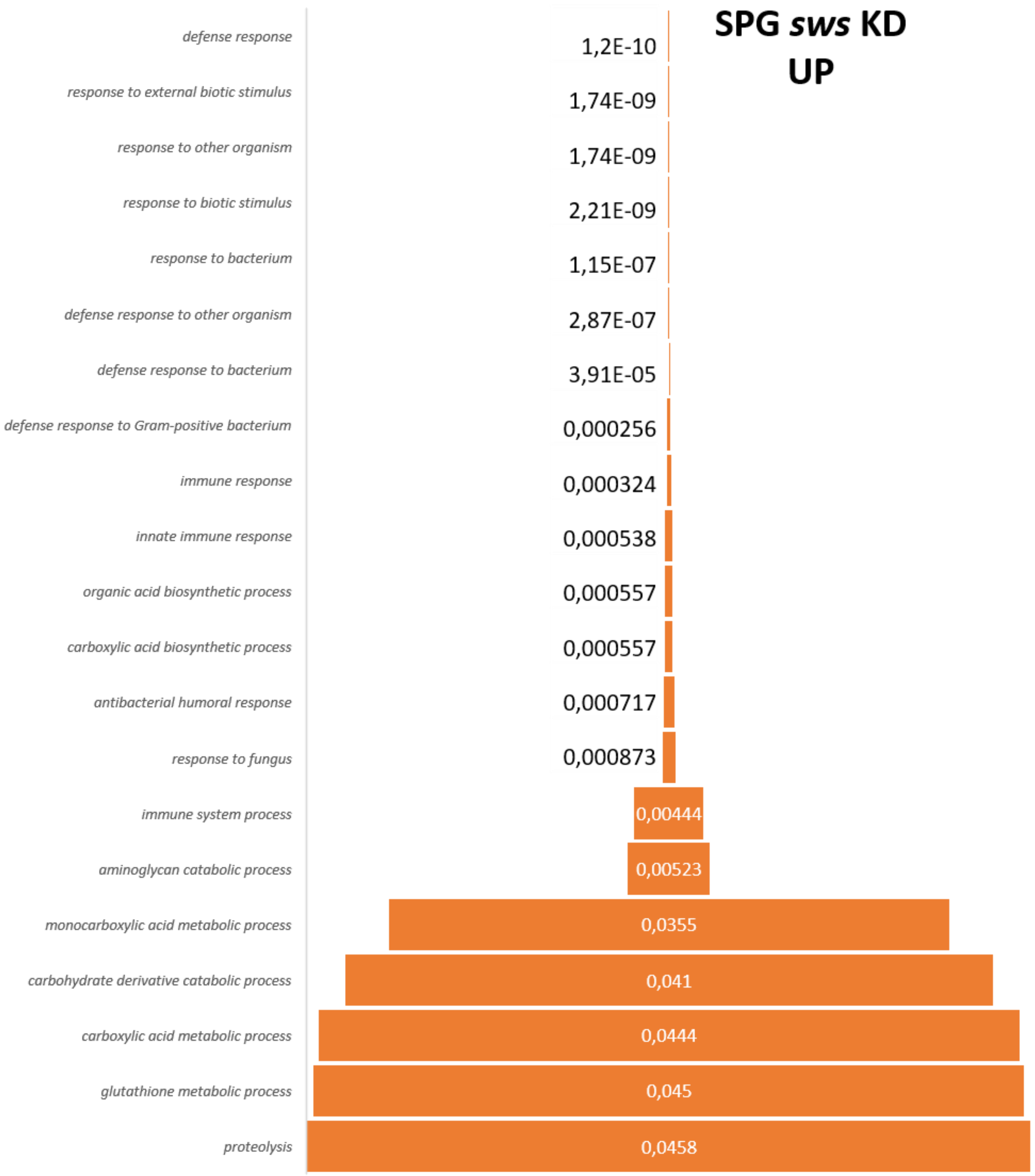

Figure S4. Processes under control of upregulated genes in 30-day-old SPG *sws* KD males compared to *CantonS* control and respective FDR-adjusted p-values of functional enrichment analysis in g:Profiler software.

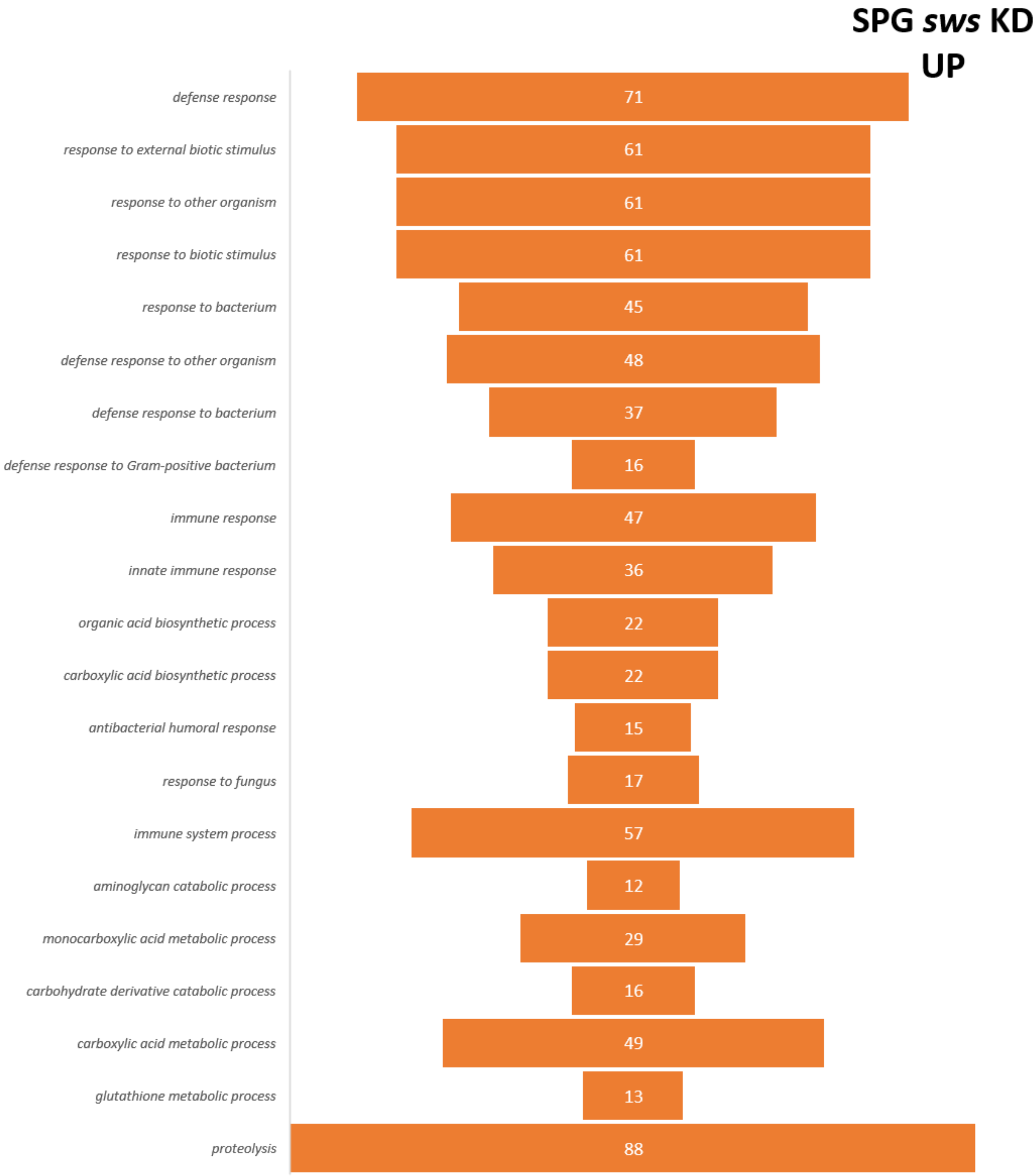

Figure S5. Processes under control of upregulated genes in 30-day-old SPG *sws* KD males compared to *CantonS* control and respective query gene number from functional enrichment analysis in g:Profiler software.

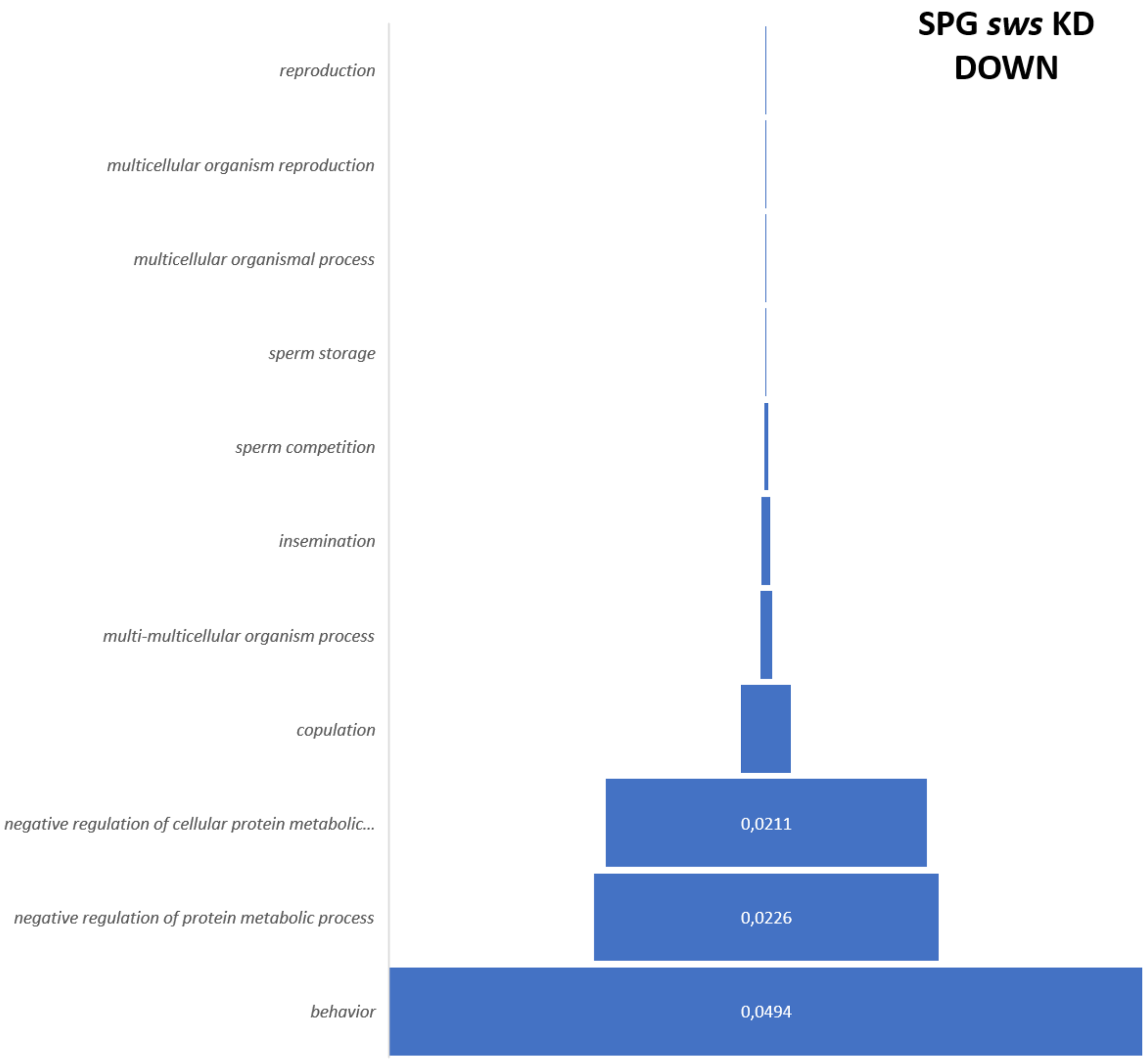

Figure S6. Processes under control of downregulated genes in 30-day-old SPG *sws* KD males compared to *CantonS* control and respective FDR-adjusted p-values of functional enrichment analysis in g:Profiler software.

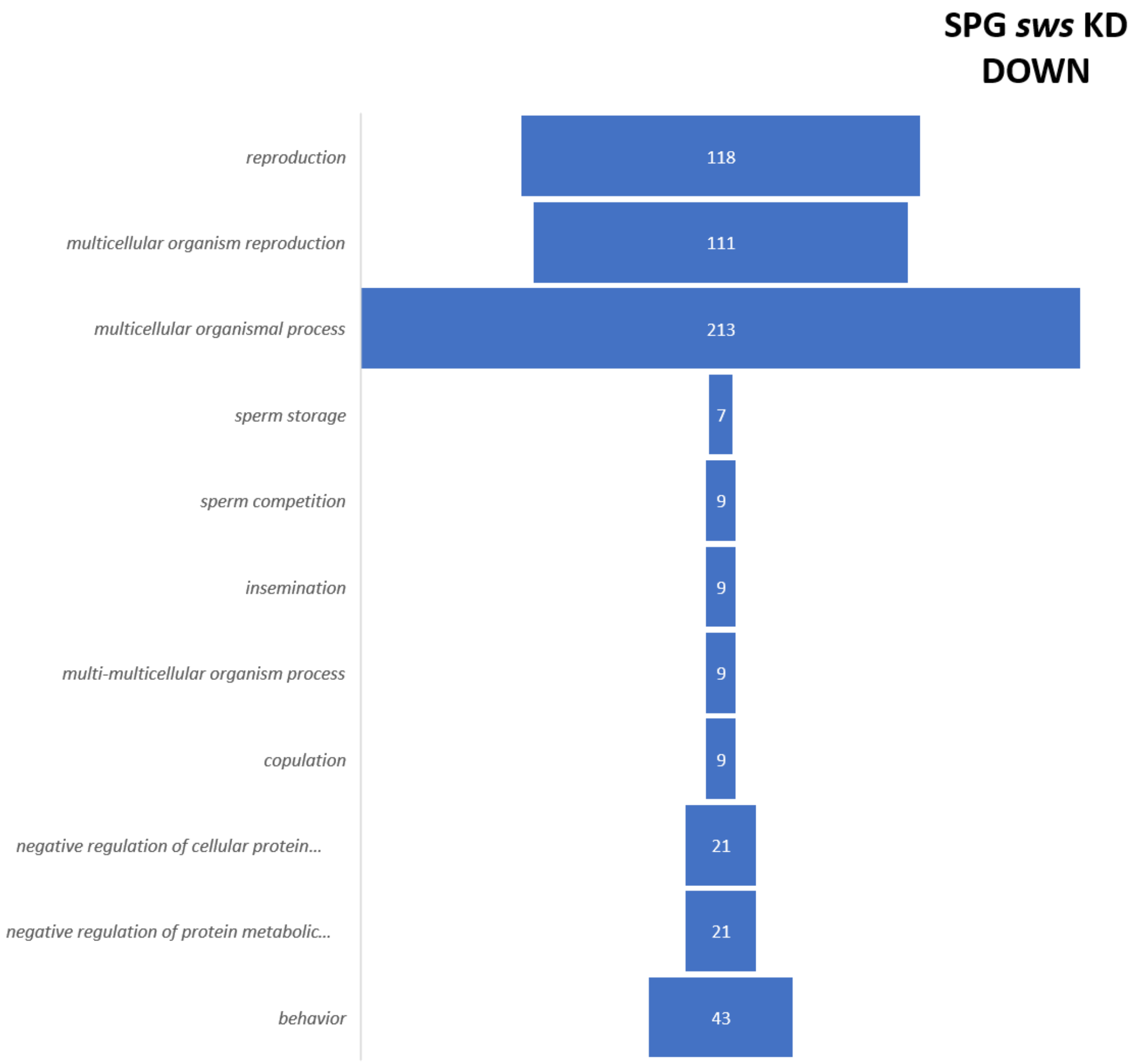

Figure S7. Processes under control of downregulated genes in 30-day-old SPG *sws* KD males compared to *CantonS* control and respective query gene number from functional enrichment analysis in g:Profiler software.

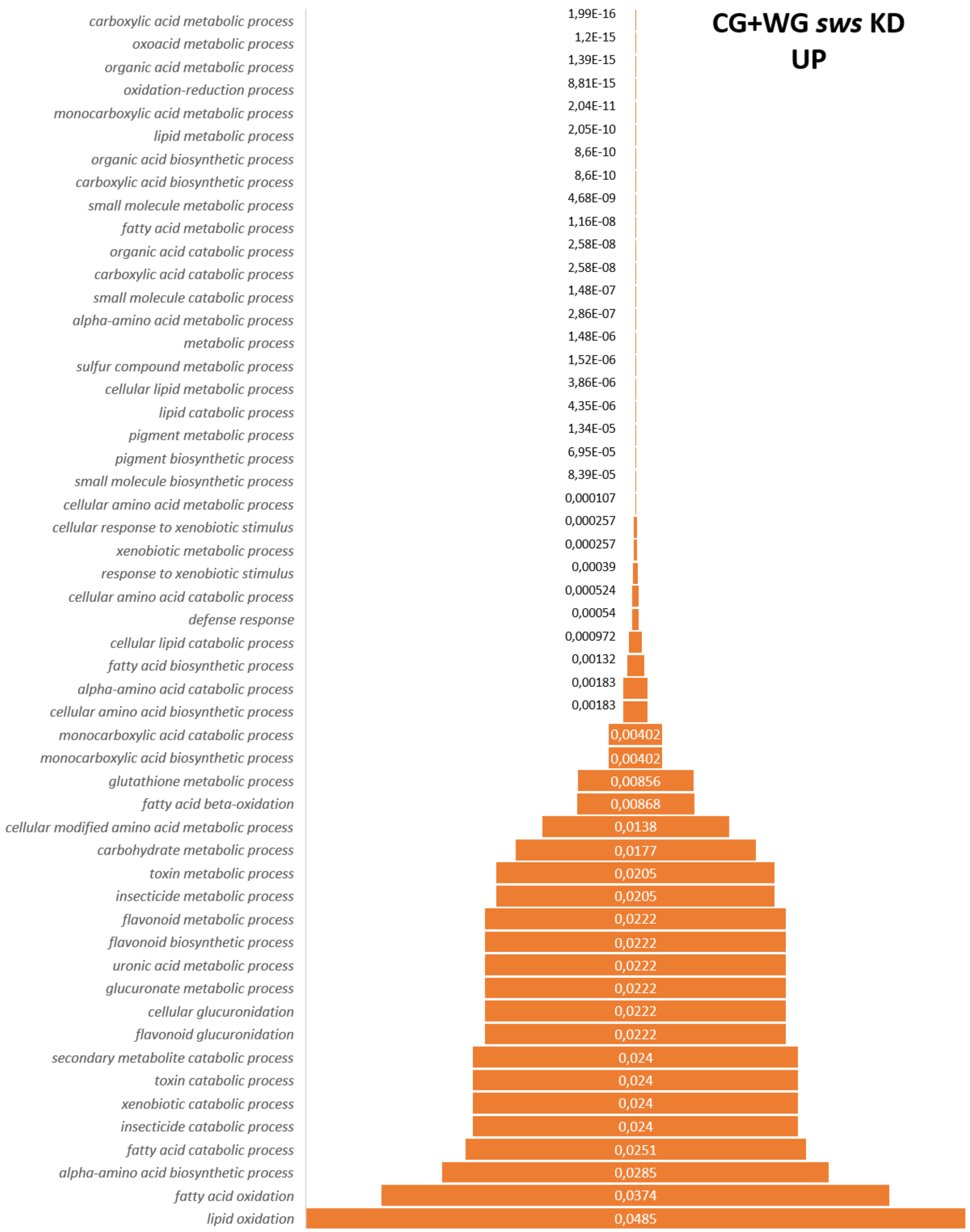

Figure S8. Processes under control of upregulated genes in 30-day-old CG+WG *sws* KD males compared to *CantonS* control and respective FDR-adjusted p-values of functional enrichment analysis in g:Profiler software.

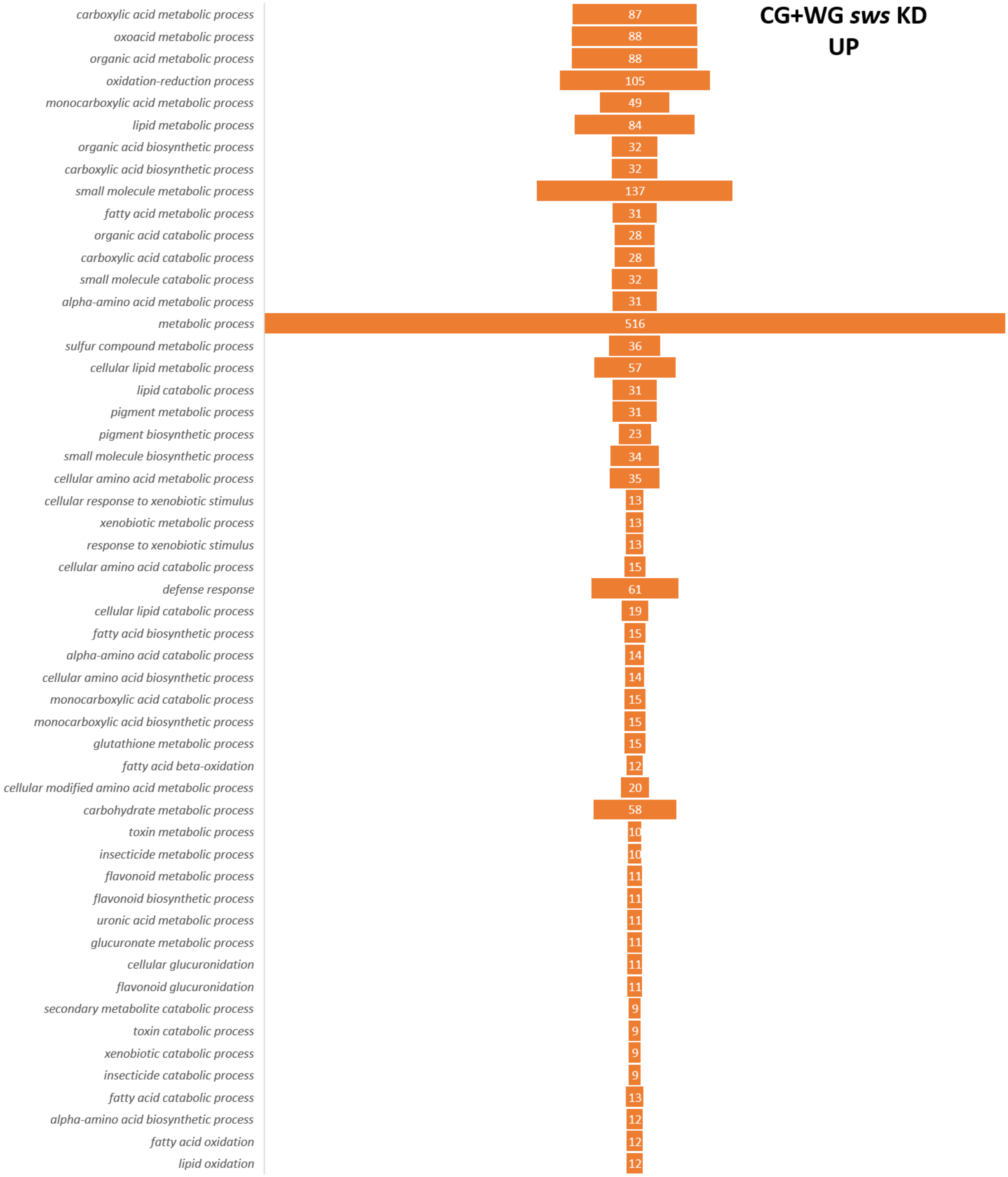

Figure S9. Processes under control of upregulated genes in 30-day-old CG+WG *sws* KD males compared to *CantonS* control and respective query gene number from functional enrichment analysis in g:Profiler software.

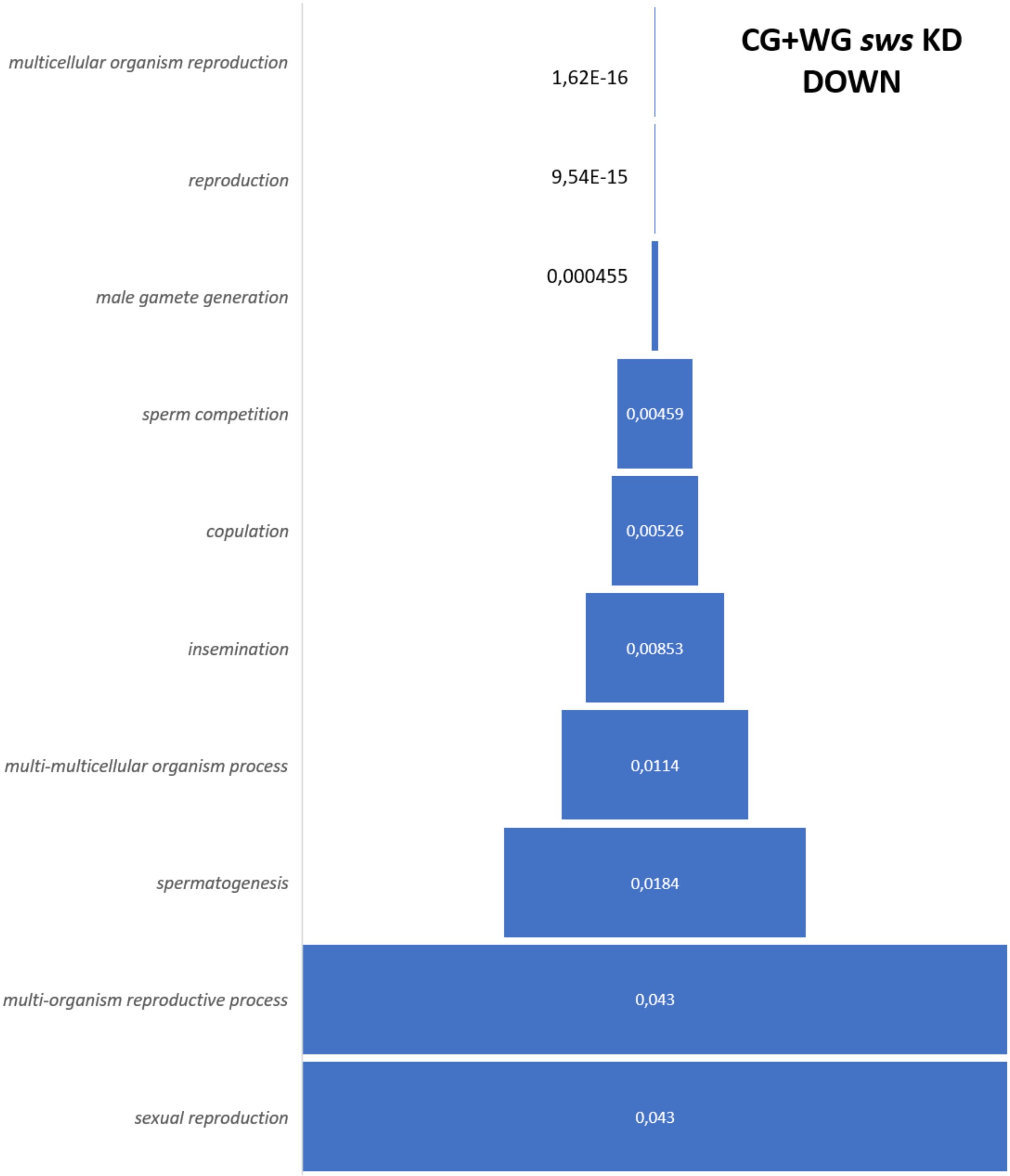

Figure S10. Processes under control of downregulated genes in 30-day-old CG+WG *sws* KD males compared to *CantonS* control and respective FDR-adjusted p-values of functional enrichment analysis in g:Profiler software.

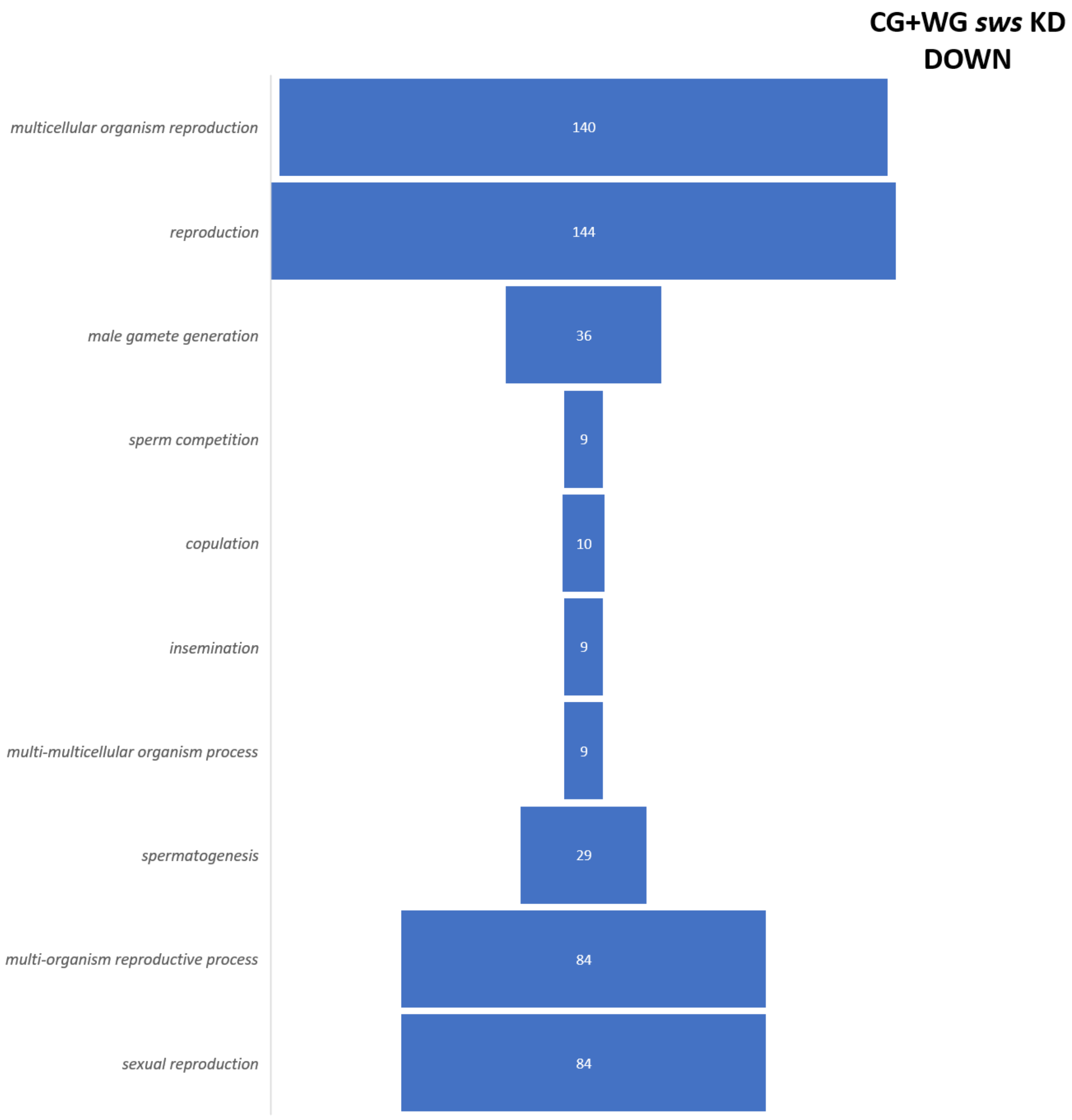

Figure S11. Processes under control of downregulated genes in 30-day-old CG+WG *sws* KD males compared to *CantonS* control and respective query gene number from functional enrichment analysis in g:Profiler software.

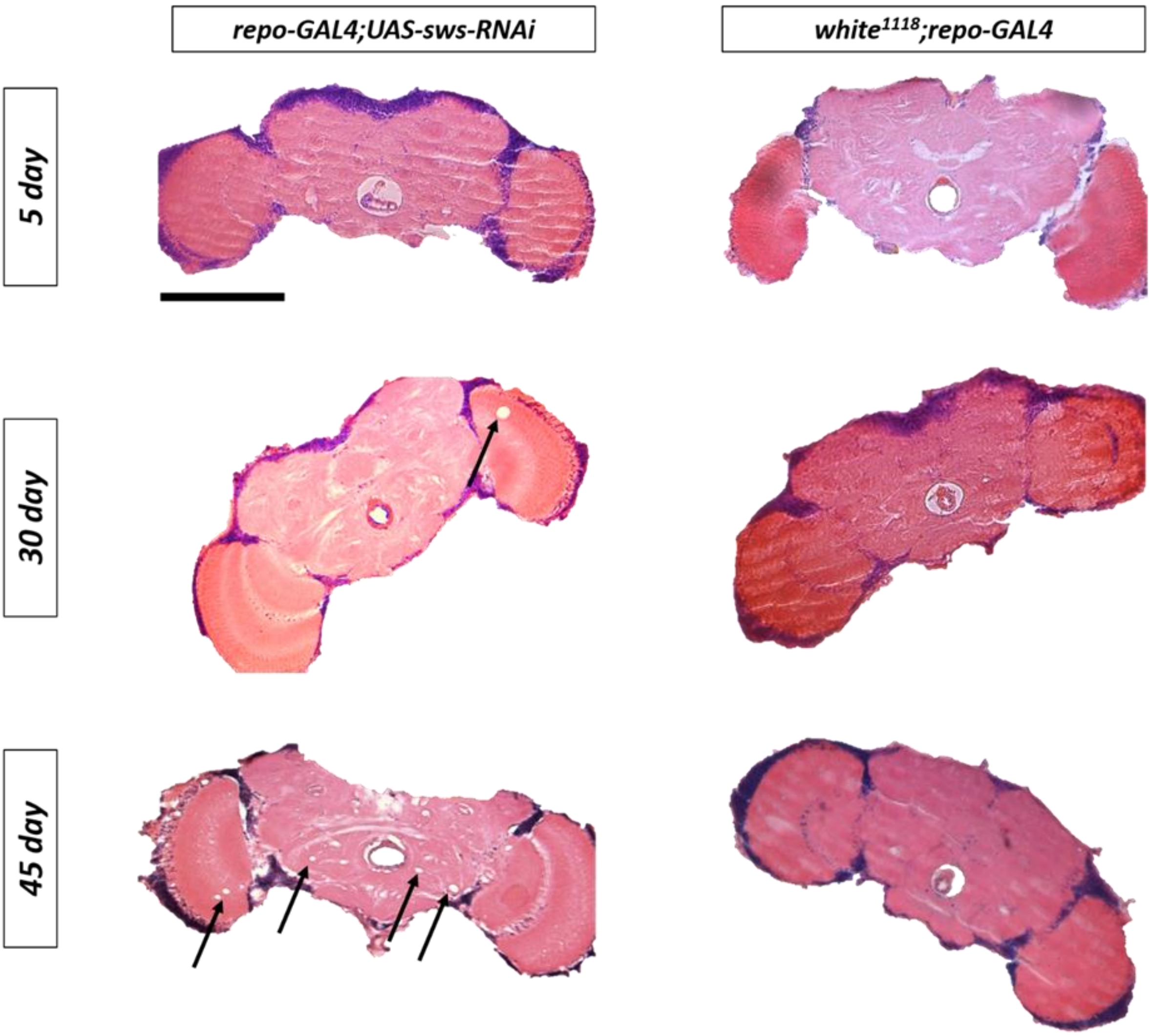

150 µm.

Figure S12. The typical images of H&E stained paraffin brain sections of *sws* KD brain (*repo-GAL4;UAS-sws-RNAi*) males and respective F1 males from crossing w^{1118} females and *repo-GAL4* males (*white*^{1118}*;repo-GAL4*) of different age (5, 30, 45 day of imago's life, +25 °C). Vacuoles (areas with no tissue due to neurodegeneration) are marked with arrows. Scale bar: 150 µm.
